# A Case of a Novel Perforin Gene Variant in Severe Familial Hemophagocytic Lymphohistiocytosis Type 2 (FHL2)

**DOI:** 10.1155/crh/1949986

**Published:** 2026-03-09

**Authors:** Hiroshi Yamauchi, Moeko Hino, Kazuyuki Meguro, Taiji Nakano, Takahiro Aoki, Yoshiharu Yamashita, Tomoko Okunushi, Takeshi Yamamoto, Hironori Sato, Takahiro Yasumi, Yuiko Hirata, Hirofumi Shibata, Hiroshi Nakajima, Hiromichi Hamada

**Affiliations:** ^1^ Department of Pediatrics, Graduate School of Medicine, Chiba University, Chiba, Japan, chiba-u.ac.jp; ^2^ Clinical Genetics, Chiba University Hospital, Chiba, Japan, chiba-u.ac.jp; ^3^ Department of Allergy and Clinical Immunology, Graduate School of Medicine, Chiba University, Chiba, Japan, chiba-u.ac.jp; ^4^ Department of Pediatrics, Graduate School of Medicine, Kyoto University, Kyoto, Japan, kyoto-u.ac.jp

**Keywords:** cyclosporine, etoposide, familial hemophagocytic lymphohistiocytosis Type 2, liposteroid, nonmyeloablative conditioning, perforin

## Abstract

**Introduction:**

Hemophagocytic lymphohistiocytosis (HLH) is a life‐threatening hyperinflammatory syndrome caused by excessive cytokine release from activated T cells and macrophages. Primary HLH, or familial HLH (FHL), results from genetic mutations affecting cytotoxic lymphocyte function.

**Case Report:**

We present a case of FHL Type 2 (FHL2) caused by compound heterozygous variants in the PRF1 gene, including one novel missense variant of p.Ala21Val (A21V). A 5‐month‐old boy presented with persistent fever, pancytopenia, coagulopathy, hepatosplenomegaly, and elevated ferritin, meeting the HLH‐2004 diagnostic criteria. Bone marrow revealed hemophagocytosis, and NK cell activity was markedly reduced. Genetic analysis identified compound heterozygous PRF1 variants: A21V and p.Pro16Ser (P16S). Flow cytometric analysis demonstrated markedly reduced PRF1 protein expression in the patient’s NK cells. The patient was treated with etoposide, dexamethasone palmitate, and cyclosporine, followed by cord blood transplantation. The patient has been in remission for over a year.

**Discussion:**

The PRF1 A21V variant has not been described in the public database or the literature and is therefore considered a novel pathogenic variant for FHL2 with functional validation. Although the PRF1 P16S variant has been previously reported in the heterozygous state in an adult patient with primary HLH, our findings provide functional and clinical evidence supporting a contributory role of the P16S variant in autosomal recessive early‐onset FHL2 when present in trans with the novel A21V variant.

**Conclusion:**

We identified a previously unreported PRF1 variant, A21V, and provided the first functional evidence of impaired perforin expression associated with A21V/P16S, highlighting the importance of functional validation of rare PRF1 variants in FHL2.

## 1. Introduction

Hemophagocytic lymphohistiocytosis (HLH) is a fatal disease characterized by excessive inflammatory cytokine production [1]. Primary HLH is caused by genetic factors, and multiple causative variants have been identified [2–10]. The PRF1 gene encodes perforin, a major component of cytotoxic granules. Abnormal PRF1 is an important factor in primary HLH [2–5, 9, 10]. Although numerous PRF1 variants have been reported, interpretation of rare missense variants remains challenging, especially when the variant is unreported or functional evidence is lacking. Here, we report a patient with severe early‐onset familial HLH (FHL) Type 2 (FHL2) caused by compound heterozygous PRF1 variants, including one novel missense variant. This case highlights the importance of integrating genomic and functional analyses to clarify the pathogenicity of rare PRF1 variants.

## 2. Case Presentation

A 5‐month‐old boy was admitted with a chief complaint of fever. He was born at 38 weeks and 5 days of gestation without perinatal complications, with a birth weight of 3062 g. He was the first child of nonconsanguineous parents and had a twin sibling who had died in utero at 6 months of gestation.

Five days before admission, the patient developed a fever accompanied by malfeeding. Two days before admission, his condition worsened, with frequent watery stools and progressive lethargy, suggesting a systemic inflammatory response. His previous physician suspected HLH caused by Epstein–Barr virus (EBV) infection and referred him to our hospital.

On admission, body temperature was 38.6°C, blood pressure 95/50 mmHg, and oxygen saturation 95% on nasal cannula oxygen at 0.5 L/min. He appeared lethargic with poor vitality. His skin showed reticular, erythematous patches throughout the body. Breath sounds were markedly diminished bilaterally. The liver was palpable 3.0 cm below the right costal margin; the spleen was not palpable, possibly due to fluid accumulation. Bilateral leg edema was evident.

Laboratory tests on admission revealed pancytopenia; prolonged prothrombin time; hypofibrinogenemia; elevated D‐dimer level, meeting the diagnostic criteria of disseminated intravascular coagulation (DIC); severely decreased NK cell activity; and marked hyperferritinemia. Soluble IL‐2 receptor levels were elevated, a hallmark of HLH‐driven immune activation. Bone marrow examination showed hemophagocytosis, confirming excessive immune activation and increased phagocytic activity (Table 1). Contrast‐enhanced computed tomography showed hepatosplenomegaly and pronounced enhancement of the hepatic periportal region. Moderate pleural effusion was also present.

**TABLE 1:** Laboratory findings at the time of admission.

Parameter	Result	Normal range (if available)
WBC	2700/μL	5000–10,000/μL
RBC	268 × 10^4^/μL	410–530 × 10^4^/μL
Hb	7.2 g/dL	13.0–17.0 g/dL
Plt	1.1 × 10^4^/μL	13–35 × 10^4^/μL
Band	1.5%	0%–5%
Seg	0%	40%–70%
Eos	0%	1%–4%
Baso	0%	0%–1%
Mono	3.5%	2%–8%
Lymph	78%	20%–40%
Reac‐Lymph	17%	0%–5%
TP	3.8 g/dL	6.5–8.0 g/dL
Alb	2.4 g/dL	4.0–5.0 g/dL
CK	665 U/L	45–163 U/L
AST	367 U/L	13–30 U/L
ALT	267 U/L	10–42 U/L
LD	1477 U/L	120–240 U/L
Cre	0.2 mg/dL	0.6–1.1 mg/dL
UN	6 ng/dL	8–20 mg/dL
TG	288 mg/dL	< 150 mg/dL
Na	130 mmol/L	135–145 mmol/L
K	3.5 mmol/L	3.6–5.0 mmol/L
CRP	1.18 mg/dL	< 0.3 mg/dL
FER	5403 ng/mL	20–200 ng/mL
APTT	32.4 s	25–35 s
PT‐INR	1.9	0.9–1.1
Fib	51 mg/dL	200–400 mg/dL
FDP	6 mg/mL	< 5 μg/mL
D‐dimer	2.7 mg/mL	< 1.0 μg/mL
AT‐III	44%	80%–120%
IgG	214 mg/dL	861–1747 mg/dL
C3	92 mg/dL	79–152 mg/dL
C4	28 mg/dL	16–38 mg/dL
sIL‐2R	25,995 U/mL	121–613 U/mL
BM‐NCC	48,000/μL	10,000–20,000/μL
BM‐MgK	12/μL	2–10/μL
BM‐M/E	2.1	1.5–3.3
NK cell activity	8%	18%–40%
CMV‐IgM	0.2 (−)	Negative
EBV‐IgM	0.2 (−)	Negative
HSV‐IgM	0.2 (−)	Negative

*Note:* WBC, white blood cell count; RBC, red blood cell count; Hb, hemoglobin; Plt, platelet count; Band, band neutrophils; Seg, segmented neutrophils; Eos, eosinophils; Baso, basophils; Mono, monocytes; Lymph, lymphocytes; Reac‐Lymph, reactive lymphocyte; Alb, albumin; AST, aspartate aminotransferase; ALT, alanine aminotransferase; Cre, creatinine; TG, triglyceride; Na, sodium; K, potassium; FER, ferritin; Fib, fibrinogen; AT‐III, Antithrombin III; IgG, immunoglobulin G; C3, Complement component 3; C4, Complement component 4; sIL‐2R, Soluble interleukin‐2 receptor; MgK, megakaryocyte count; M/E, myeloid/erythroid ratio; CMV‐IgM, cytomegalovirus IgM.

Abbreviations: APTT, activated partial thromboplastin time; CK, creatine kinase; CRP, C‐reactive protein; D‐dimer, D‐dimer; EBV‐IgM, Epstein–Barr virus IgM; FDP, fibrin degradation product; HSV‐IgM, herpes simplex virus IgM; LD, lactate dehydrogenase; NCC, nucleated cell count; NK cell activity, natural killer cell activity; PT‐INR, prothrombin time–international normalized ratio; TP, total protein; UN, urea nitrogen.

Genetic analysis using the HLH panel revealed that the patient had compound heterozygous PRF1 variants, A21V from his father and P16S from his mother (Figures 1(a) and 1(b)). Defective perforin expression in NK cells was confirmed (Figure 1(c)).

FIGURE 1Identification and functional characterization of PRF1 variants in a patient with FHL2. (a) Patient pedigree and PRF1 genotyping. Filled symbol, affected; open symbols, unaffected. (b) Details of the two missense PRF1 variants identified in the patient. (c) Flow cytometry of the perforin protein expression in NK cells from the patient and an age‐matched control stained for CD56 versus perforin. CD3^−^CD56^+^ population was analyzed.

**Figure:**
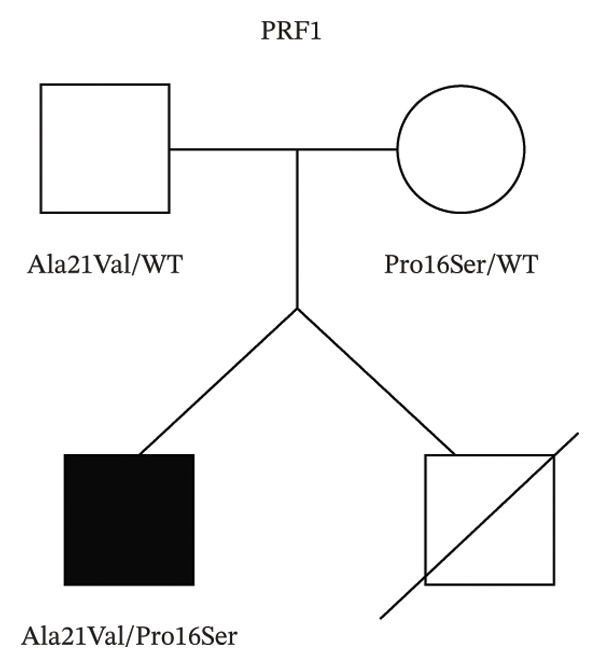
(a)

**Figure:**
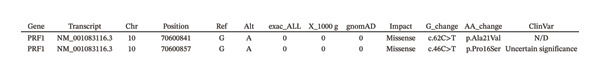
(b)

**Figure:**
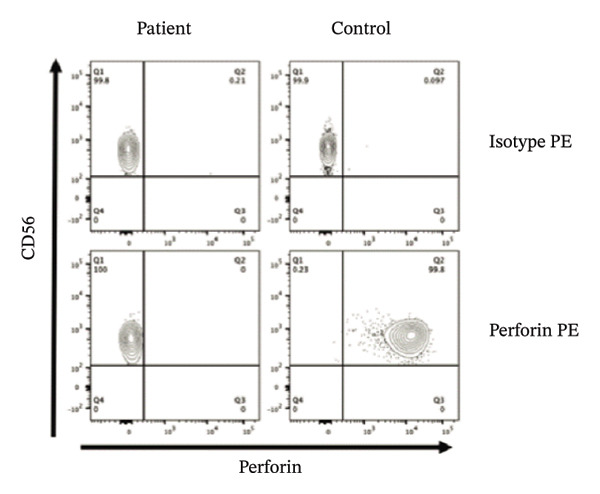
(c)

The patient met the HLH‐2004 diagnostic criteria based on laboratory findings at admission and was diagnosed with FHL [1]. Treatment with etoposide and liposomal steroid (dexamethasone palmitate) was initiated, and cyclosporine was added after two weeks of initial therapy, consistent with current clinical practice based on the HLH‐2004 protocol [11]. He subsequently underwent nondestructive bone marrow conditioning with thymoglobulin, fludarabine, and melphalan, followed by cord blood transplantation on Day 49 after the onset of illness. He has since survived without any FHL symptoms for over a year.

## 3. Discussion

In this study, we report a clinically typical case of FHL2 and provide functional characterization of two PRF1 variants, including a novel variant, A21V, and a previously reported variant in the context of heterozygous form in an adult HLH patient [12], P16S, thereby extending the genotype–phenotype correlation of PRF1‐associated FHL2.

HLH is characterized by persistent hypercytokinemia resulting from overactivated T cells and macrophages, leading to abnormal histiocyte activation and hemophagocytosis [2–4]. Upon recognition of target cells such as tumor cells and virus‐infected cells, cytotoxic T lymphocytes (CTLs) and NK cells release cytotoxic granules to target cells. Perforin, an effector molecule contained in cytotoxic granules, forms small pores in the target cell membrane to induce apoptosis. Impaired target cell killing results in persistent stimulation of CTLs and NK cells and proinflammatory cytokine overproduction that causes secondary hyperactivation of macrophages.

FHL is currently classified into five subtypes (FHL1–FHL5), all characterized by reduced cytotoxic activity due to defective perforin activity or impaired transport or release of perforin‐containing cytotoxic granules [2–4]. Although the causative genetic variants for FHL1 and the pathogenicity of FHL1 remain unidentified, the underlying genetic mechanisms for FHL2–FHL5 have been established. The current patient was diagnosed with FHL2, caused by genetic defects in the PRF1 gene encoding perforin. Therefore, perforin expression is deficient, attenuated, or functionally impaired [9].

Consistent with previous reports describing diverse PRF1 variant spectra across different ethnic groups [10], our patient exhibited a typical clinical presentation of FHL, and compound heterozygous missense variants in PRF1 were identified. The PRF1 A21V variant is currently not described in major databases, including gnomAD, ToMMo, ClinVar, HGMD, and dbSNP. However, our functional analysis showed defective PRF1 protein expression, consistent with the in silico predictions of pathogenicity using SIFT, PolyPhen‐2, and M‐CAP. Based on the patient’s typical clinical presentation and the results of functional analysis, we identified a novel PRF1 missense variant A21V as a pathogenic variant for FHL2.

The PRF1 P16S variant is currently listed in ClinVar as a variant of “Uncertain significance” and is also reported in dbSNP (rs1031606439). Although P16S has not been reported as a causative variant of AR form of FHL2, Jin et al. previously identified this variant in adult‐onset HLH patients in a heterozygous state and showed decreased PRF1 protein expression [12]. This variant was not found in the MAF, ToMMo, or gnomAD databases but was reported in dbSNP (rs1031606439). In silico analyses using SIFT, PolyPhen‐2, and M‐CAP also suggest its pathogenic potential, as evidenced by a M‐CAP score of 0.076 and classification as “Possibly Pathogenic.” Based on these findings, along with the patient’s clinical presentation and the functional analysis, we also position PRF1 P16S as a pathogenic variant causing AR form FHL2.

In conclusion, A21V is a novel PRF1 variant, and this case supports a pathogenicity of the previously reported P16S variant in autosomal recessive early‐onset FHL2 when present in trans with the A21V variant.

## Author Contributions

Hiroshi Yamauchi: conceptualization, investigation, and writing–original draft.

Kazuyuki Meguro and Moeko Hino: supervision and writing–review and editing.

Taiji Nakano: validation.

Tomoko Okunushi, Hironori Sato, Yoshiharu Yamashita, and Takeshi Yamamoto: resources.

Takahiro Yasumi: methodology and validation.

Yuiko Hirata and Hirofumi Shibata: investigation.

Hiroshi Nakajima, Takahiro Aoki, and Hiromichi Hamada: writing–review and editing.

## Funding

This research received no funding.

## Disclosure

A preprint of this manuscript has previously been published: Hiroshi Yamauchi, et al. A case of a novel perforin gene variant in severe familial hemophagocytic lymphohistiocytosis Type 2 (FHL2) [13].

## Ethics Statement

Although this study involves the off‐label use of certain drugs, their use was reviewed and approved by the Chiba University Ethics Committee (Approval number: 01‐06).

## Consent

Written informed consent was obtained from the patient’s guardians after a thorough explanation of the study. Ethical considerations were prioritized, and great care was taken to ensure that the privacy of the patients was protected.

## Conflicts of Interest

The authors declare no conflicts of interest.

## Data Availability

The data supporting the findings of this study are not publicly available due to patient privacy concerns.
